# Novel approach to defining major abdominal surgery

**DOI:** 10.1093/bjs/znad355

**Published:** 2023-11-13

**Authors:** Alona Courtney, Yasmin Dorudi, Jonathon Clymo, Daria Cosentino, Timothy Cross, Suneetha Ramani Moonesinghe, Sina Dorudi

**Affiliations:** Department of Targeted Intervention, Division of Surgery and Interventional Sciences, University College London, London, UK; The Princess Grace Hospital, HCA Healthcare UK, London, UK; University of Bristol Medical School, Bristol, UK; Imperial College Healthcare NHS Trust, St Mary’s Hospital, London, UK; Clinical Operations Group, HCA Healthcare UK, London, UK; Clinical Operations Group, HCA Healthcare UK, London, UK; Department of Targeted Intervention, Division of Surgery and Interventional Sciences, University College London, London, UK; Department of Targeted Intervention, Division of Surgery and Interventional Sciences, University College London, London, UK; The Princess Grace Hospital, HCA Healthcare UK, London, UK

## Introduction

Over 5.8 million abdominal operations and procedures were recorded in England between April 2021 and March 2022^1^. Although a definition of major surgery has been proposed by the Delphi consensus among European Surgical Association members^2^, there is no clear consensus regarding which surgical procedures constitute major abdominal surgery (MAS). Despite this, multiple sources in the literature, including perioperative morbidity and mortality scoring systems, national audits, and private healthcare coding schedules^3–5^, have alluded to this type of surgery without any underlying qualification. To clarify this area, a scoping literature review was conducted to derive a definition of MAS, based on content analysis of the terminology used to describe major abdominal surgical procedures^6^. MAS was defined as an intraperitoneal operation with no primary involvement of the thorax, involving either luminal resection and/or resection of a solid organ associated with the gastrointestinal tract. The aim of the current study was to verify the discriminative ability of this hypothesized definition of MAS using real-world data analysis and unsupervised machine learning.

## Methods

The discriminative properties of the narrative definition were scrutinized using two independent tests: real-world data analysis and unsupervised machine learning. To facilitate this process, the narrative definition was translated into procedure codes listed within sections 11 (abdomen, excl. urinary & reproductive organs) and 14 (female reproductive organs) of the Clinical Coding & Schedule Development (CCSD)^5^. Of the 337 codes, 58 were excluded as these described non-operative procedures, endoscopy, investigations, and radiotherapy (*Table S1* and *S2*). The real-world data were extracted from the electronic health records of seven hospitals within the HCA Healthcare UK group over 5 years (1 April 2017 to 31 March 2022).

The following proxy measures were employed: median patient age, median procedure duration, median duration of hospital stay, and proportion of patients admitted to critical care after operation. These proxy measures were collected routinely for all patients and represented the most complete data set. Other proxy measures (return to theatre, perioperative transfusion rate, and unplanned readmission) did not have discriminative ability within the algorithm, likely because of insufficient case volume. Further details on data extraction can be found in the *supplementary material*. The real-world data analysis involved comparison of the MAS and non-MAS cohorts against defined proxy measures. These cohorts were based on translation of the narrative definition into CCSD procedure codes, resulting in the following split: 84 MAS codes and 195 non-MAS codes.

Unsupervised machine learning, using k-medoids clustering algorithm (partitioning around medoids) with Euclidian distance within the R statistical computing environment (R Project for Statistical Computing, Vienna, Austria)^7^, was used to cluster all procedure codes into MAS and non-MAS cohorts based on the defined proxy measures. A k-medoids clustering algorithm was chosen because it suited the objective of identifying the most representative procedure within the MAS and non-MAS cohorts. Further details about the machine learning algorithm methodology can be found in the *supplementary material*. Statistical analysis was performed using open-source software JASP Team version 0.16.4, which has been verified extensively against other software^8^.

## Results

Data extraction from electronic patient records returned 16 353 procedures over a consecutive 5-year interval, equivalent to 73 MAS codes and 141 non-MAS codes. Based on this codified narrative definition, there was a statistically significant difference between the MAS and non-MAS cohorts, when all proxy measures were compared (*P* < 0.001 for all values) (*Table 1*).

**Table 1:** Differences between patient cohorts

	n	Procedure codes (n)	Age (years)	Procedure duration (min)	Length of hospital stay (days)	Rate of ICU admission (%)
**Real-world data analysis**						
MAS	3880	73	59 (55–65)	189 (145–268)	8 (6–10)	78 (59–92)
Non-MAS	12 473	141	51 (41–60)	70 (42–125)	1 (0–5)	4 (0–27)
**Unsupervised machine learning**						
Cluster 1 (MAS)	3628	51	60 (57–65)	190 (139–258)	9 (7–11)	79 (58–89)
Cluster 2 (non-MAS)	12 408	76	50 (43–54)	58 (39–96)	1 (0–2)	4 (0–12)

Values are median (i.q.r.). MAS, major abdominal surgery.

**Fig. 1:**
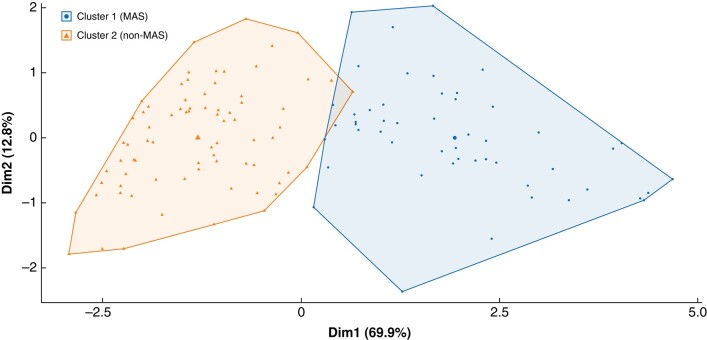
Partitioning around medoids algorithm clusters for abdominal surgery procedures Partitioning around medoids algorithm clusters for abdominal surgery procedures

Interrogation of the same data set using the unsupervised machine learning clustering algorithm resulted in the following split: 51 codes were allocated into cluster 1 and 76 codes into cluster 2. The medoid for cluster 1 was reversal of Hartmann’s procedure (CCSD code H3390); this was considered representative of the MAS procedures. The medoid for cluster 2 was open repair of incisional hernia not requiring mesh (CCSD code T2500), aligning well with the non-MAS procedures (sensitivity 85.7 per cent, specificity 88.5 per cent). Two-dimensional algorithm clusters, using principal component analysis, can be visualized in *Fig. 1*. Comparison of the proxy measures discerned significant differences between cluster 1 (considered MAS) and cluster 2 (considered non-MAS) (*P* < 0.001 for all parameters) (*Table 1*). Overall, the percentage agreement between the real-world data analysis and unsupervised machine learning classifications was 87.4 per cent (Cohen’s κ 0.736) (*Table S3*). Some 152 of 279 CCSD codes involved procedures with fewer than 10 patients and were excluded from this exercise.

There was some discordance between the translated narrative definition (real-world data analysis) and unsupervised machine learning with regard to 16 codes. Specifically, seven codes defined by the narrative definition as MAS were distributed by the algorithm to the non-MAS cluster 2, and nine codes defined by the narrative definition as non-MAS were distributed by the algorithm to the MAS cluster 1 (*Table S4*). However, further analysis revealed that the seven MAS procedure codes grouped into cluster 2 (non-MAS) by the algorithm were all upper gastrointestinal operations (at least half were bariatric), with the exception of cytoreductive surgery for ovarian malignancy. Patients in this cohort were younger and recovered more quickly after surgery. There was a significant difference in all proxy measures compared with cluster 1 (MAS) (*P* ≤ 0.020). The nine non-MAS codes (*Table S4*) discordantly classified into cluster 1 (presumed MAS) by the algorithm were significantly different in all proxy parameters compared with cluster 2 (presumed non-MAS) (*P* ≤ 0.030). Specifically, these patients underwent longer procedures, had a longer postoperative hospital stay, and had higher rate of critical care admissions after surgery.

## Discussion

This is the first study to verify scientifically the discriminative properties of a hypothesized definition of MAS using real-world data and unsupervised machine learning. Both methods ascertained a significant difference in all proxy measures when MAS procedures were compared with non-MAS procedures. Specifically, the study confirmed the hypothesis that patients undergoing MAS tended to be older, and had a longer operation and postoperative hospital stay. The risk of being admitted to critical care was 19 times greater for patients undergoing MAS procedures. These findings are unsurprising, given the impact of (gut) luminal and solid-organ resection on patients. Operative tissue trauma (ensuing from tissue retraction, thermal injury, intraperitoneal organ and peritoneal dissection, and bowel mobilization) results in catabolic stress, provokes an inflammatory response, and affects the microbiome, all of which increase the probability of postoperative complications and delay restoration of normal organ function^9–11^.

The exclusion of a significant number of codes from the clustering algorithm may constitute a limitation of this study, because the allocation into each cluster depended on the position of other variables in the mix. However, the observations of a significant difference in the proxy measures and a high degree of concordance between the real-world data and unsupervised machine learning provide reassurance of the veracity of the hypothesized definition of MAS.

Interestingly, this definition of MAS is at odds with the definition of major surgery proposed by Martin *et al.*^2^. The latter was based entirely on patient- and procedure-based criteria, both of which risk the introduction of bias and confounding factors when selecting MAS procedures as they are patient- and operator-dependent. Some of the criteria in the definition of Martin *et al.*^2^ (significant patient co-morbidity, organ ischaemia, postoperative metabolic stress response, high vasopressor use) were entirely absent from the themes identified by the scoping review. In addition, other criteria (long duration of operation, blood loss exceeding 1000 ml, 30-day morbidity rate over 30 per cent, mortality rate above 2 per cent, and the need for intermediate or intensive care) constituted only a small proportion of all identified themes (a mere 107 of the total of 1434 references coded).

Ultimately, further work is required to validate the definition presented in this study in a larger data set. An alternative procedure coding system, such as ICD-10-PCS, may need to be employed.

## Supplementary Material

znad355_Supplementary_Data

## Data Availability

Data reported in this paper cannot be found on publicly available databases.
