# Supplementary material for: Novel approach to defining major abdominal surgery
Source: Br J Surg. 2023 Nov 13;111(1):znad355. doi: 10.1093/bjs/znad355 (PMC10771131; doi:10.1093/bjs/znad355)
Supplement: znad355_Supplementary_Data [file znad355_supplementary_data.docx]

**Title: A novel approach to define major abdominal surgery**

Authors

Alona Courtney^1,2^, Yasmin Dorudi^3^, Jonathon Clymo^4^, Daria Cosentino^5^, Timothy Cross^5^, Suneetha Ramani Moonesinghe^1^, Sina Dorudi^1,2^

**^1^**Department of Targeted Intervention, Division of Surgery & Interventional Sciences, University College London, 3rd floor, Charles Bell House, 43-47 Foley Street, LONDON, W1W 7TS

^2^The Princess Grace Hospital, HCA Healthcare UK 42-52 Nottingham Place, London, W1U 5NY

^3^University of Bristol Medical School, First Floor, 5 Tyndall Avenue, Bristol, BS8 1UD

^4^Imperial College Healthcare NHS Trust, St Mary’s Hospital, Praed Street, London, W2 1NY

^5^Clinical Operations Group, HCA Healthcare UK, 2 Cavendish Square, London, W1G 0PU

**Corresponding author**

Alona Courtney MBChB BSc(Hons) MSc MRCS

The Princess Grace Hospital, HCA Healthcare UK, 42-52 Nottingham Place, London, W1U 5NY

**ORCID ID: 0000-0002-0077-1994**

**Supplementary Materials - Index**

| **Supplementary Methods** |  |
| --- | --- |
| Data extraction | *page 3* |
| Machine learning algorithm | *page 3* |
| **Supplementary Figures and Tables** |  |
| Table S1. CCSD codes included and excluded from Major Abdominal Surgery (MAS) cohort as per definition. | *page 4* |
| Table S2. Reasons for allocation to non-MAS category or exclusion of CCSD codes. | *page 20* |
| Table S3. Confusion matrix comparing human and machine classification of major abdominal surgery. | *page 21* |
| Table S4. Discordance between narrative definition and unsupervised machine learning clustering. | *page 22* |
| **References** | *page 23* |

**Supplementary Methods**

*Data extraction*

Data were considered at theatre visit level, and only episodes with either (i) 1 MAS code and any number of non-MAS codes or (ii) 1 non-MAS code and 0 MAS codes were included in the final analysis. Data aggregation was performed at procedure code level to evaluate median patient age, median procedure duration, median length of stay, and proportion of patients admitted to critical care post-operatively for each procedure code. Aggregate results were then combined with the overall procedure complexity rating associated with each code. Codes with fewer than ten procedures performed in the date range were excluded. The aggregated results were scaled to give each feature a mean value of 0 and standard deviation of 1.

*Machine learning algorithm*

The k-medoids family of partitioning algorithms are commonly used within the clinical field and have experienced an increased popularity over recent years. A search in PubMed using the keyword ‘k medoids’ returned 200 publications, of which over 50% have been released in the last 3 years. In particular, the PAM algorithm has been shown to perform with greater robustness to outliers than other centroid based approaches such as k-means^1^. It is generally more memory intensive than other partitioning methods, but the sample size tested in this paper lent itself to its use.

The PAM algorithm finds k representative cluster centres (medoids) and then seeks to minimise the sum of dissimilarities between the objects in each cluster and the medoid. To test the dichotomous classification of MAS  and non-MAS, a value of k = 2 was used. The PAM algorithm returned the cluster number (1 or 2) into which each procedure code was classified, along with the procedure identified as the medoid for each cluster. To visualise the clusters returned by the PAM algorithm, Principal Component Analysis was used to enable representation of the multi-dimensional inputs in two-dimensional space.

**Supplementary Figures and Tables**

**Table S1. CCSD codes included and excluded from Major Abdominal Surgery (MAS) cohort as per definition.**

| Code | Description | MAS | Reason for inclusion/exclusion from MAS |
| --- | --- | --- | --- |
| A2720 | Proximal gastric vagotomy | No | No luminal resection |
| A2730 | Highly selective vagotomy | No | No luminal resection |
| A2780 | Vagotomy with pyloroplasty | No | No luminal resection |
| A2781 | Laparoscopic vagotomy/seromyotomy | No | No luminal resection |
| A7012 | Implantation of gastroelectrical stimulation (GES) for gastroparesis | No | No luminal resection |
| B2222 | Adrenalectomy - bilateral (open) | No | Not associated with GI tract |
| B2223 | Adrenalectomy - bilateral (laparoscopic) | No | Not associated with GI tract |
| B2224 | Robotic assisted laparoscopic adrenalectomy - bilateral | No | Not associated with GI tract |
| B2232 | Adrenalectomy - unilateral (open) | No | Not associated with GI tract |
| B2233 | Adrenalectomy - unilateral (laparoscopic) | No | Not associated with GI tract |
| B2234 | Robotic assisted laparoscopic adrenalectomy | No | Not associated with GI tract |
| BT251 | Planning for insertion and removal of radioactive agent (brachytherapy) into rectal tumour | No | Radiotherapy |
| BT252 | Insertion and removal of radioactive agent (brachytherapy) into rectal tumour | No | Radiotherapy |
| BT253 | Low energy contact X ray brachytherapy (the Papillon technique) for early stage rectal cancer | No | Radiotherapy |
| BT260 | Planning for insertion and removal of radioactive agent (brachytherapy) into carcinoma of the oesophagus, bronchus or stomach | No | Radiotherapy |
| BT270 | Insertion and removal of radioactive agent (brachytherapy) into carcinoma of the oesophagus, bronchus or stomach | No | Radiotherapy |
| G0100 | Oesophagectomy/Oesophagogastrectomy with anastomosis in chest | Yes | Luminal resection |
| G0220 | Total oesophagectomy and interposition of intestine | Yes | Luminal resection |
| G0260 | Endoscopically assisted oesophagectomy | Yes | Luminal resection |
| G0300 | Sub-total oesophagectomy with anastomosis in neck | No | Involvement of thorax |
| G0400 | Open excision of lesion of oesophagus | No | No luminal resection |
| G0500 | Bypass of oesophagus | Yes | Luminal resection |
| G0640 | Closure of bypass of oesophagus | Yes | Luminal resection |
| G0730 | Repair of congenital oesophageal atresia (+/- fistula) | No | Paediatric procedure |
| G0740 | Repair of ruptured oesophagus | No | No luminal resection |
| G0920 | Oesophagocardiomyotomy (Heller's operation) | No | No luminal resection |
| G0980 | Thorascopic oesophagogastric myotomy | No | Involvement of thorax |
| G1400 | VATS excision lesion of oesophagus | No | Not intra-peritoneal procedure |
| G1421 | Endoscopic focal ablation of dysplasia in Barrett's oesophagus | No | Endoscopy |
| G1422 | Endoscopic circumferential ablation of dysplasia in Barrett’s oesophagus | No | Endoscopy |
| G1460 | Endoscopic mucosal resection of high-grade dysplasia in Barrett's oesophagus | No | Endoscopy |
| G1900 | Rigid oesophagoscopy including any biopsy, laser or diathermy destruction of lesions | No | Endoscopy |
| G2110 | Oesophageal physiology studies (including pH measurement) | No | Investigation |
| G2120 | High resolution oesophageal manometry | No | Investigation |
| G2140 | Oesophageal manometry | No | Investigation |
| G2312 | Transthoracic repair of paraoesophageal hiatus hernia | No | Involvement of thorax |
| G2320 | Transthoracic repair of diaphragmatic hernia (acquired) | No | No luminal resection |
| G2330 | Transabdominal repair of hiatus hernia | No | No luminal resection |
| G2331 | Laparoscopic repair of hiatus hernia with anti-reflux procedure (e.g. fundoplication) | No | No luminal resection |
| G2332 | Laparoscopic Insertion of magnetic band for gastro-oesophageal reflux disease (LINX) | No | No luminal resection |
| G2333 | Robotic assisted laparoscopic repair of hiatus hernia with anti-reflux procedure (e.g. fundoplication) | No | No luminal resection |
| G2340 | Transabdominal repair of diaphragmatic hernia (excluding hiatus hernia) | No | No luminal resection |
| G2400 | Transthoracic fundoplication & gastroplasty | No | No luminal resection |
| G2402 | Transthoracic fundoplication | No | Involvement of thorax |
| G2430 | Transabdominal anti-reflux operations | No | No luminal resection |
| G2590 | Revision of anti-reflux operations | No | No luminal resection |
| G2710 | Total gastrectomy and excision of surrounding tissue | Yes | Luminal resection |
| G2800 | Partial gastrectomy | Yes | Luminal resection |
| G2810 | Partial gastrectomy and excision of surrounding tissue | Yes | Luminal resection |
| G3070 | Sleeve gastrectomy (including laparoscopic) | Yes | Luminal resection |
| G3080 | Laparoscopic gastric banding | No | No luminal resection |
| G3090 | Gastric bypass eg. Roux en Y for morbid obesity (including laparoscopic) | Yes | Luminal resection |
| G3100 | Laparoscopic biliary gastric bypass | Yes | Luminal resection |
| G3210 | Gastro-jejunostomy | No | No luminal resection |
| G3220 | Revision of gastro-jejunostomy | No | No luminal resection |
| G3400 | Gastrostomy | No | No luminal resection |
| G3440 | Closure of gastrostomy | No | No luminal resection |
| G3490 | Endoscopic removal of percutaneous endoscopic gastrostomy (PEG) tube | No | Endoscopy |
| G3520 | Closure of perforated ulcer of stomach | No | No luminal resection |
| G3580 | Laparoscopic closure of peptic ulcer | No | No luminal resection |
| G3610 | Gastropexy (for reflux) | No | No luminal resection |
| G3800 | Open operations on stomach not elsewhere classified | No | No luminal resection |
| G3870 | Laparoscopic removal of gastric band | No | No luminal resection |
| G4010 | Pyloromyotomy | No | No luminal resection |
| G4020 | Surgery for correction of congenital intestinal atresias | No | Paediatric procedure |
| G4030 | Pyloroplasty | No | No luminal resection |
| G4370 | Therapeutic oesophago-gastro-duodenoscopy (OGD) with elective banding of oesophageal varices | No | Endoscopy |
| G4410 | Therapeutic oesophago-gastro-duodenoscopy (OGD) with insertion of prosthesis | No | Endoscopy |
| G4430 | Therapeutic oesophago-gastro-duodenoscopy (OGD) with dilatation | No | Endoscopy |
| G4440 | Therapeutic oesophago-gastro-duodenoscopy (OGD) with insertion percutaneous endoscopic gastrostomy/percutaneous endoscopic jejunostomy | No | Endoscopy |
| G4442 | Gastroscopic balloon insertion | No | Endoscopy |
| G4443 | Gastroscopic balloon removal | No | Endoscopy |
| G4460 | Endoscopic radiofrequency ablation for gastro-oesophageal reflux disease | No | Endoscopy |
| G4480 | Therapeutic enteroscopy | No | Endoscopy |
| G4520 | Diagnostic enteroscopy | No | Endoscopy |
| G4530 | Catheterless oesophageal pH monitoring (e.g. Bravo) | No | Investigation |
| G4680 | Endoscopic mucosal resection (upper gastrointestinal tract) | No | Endoscopy |
| G4690 | Endoscopic submucosal dissection of gastric lesions | No | Endoscopy |
| G5000 | Open excision of lesion of duodenum | No | No luminal resection |
| G5010 | Open excision of congenital lesion of duodenum including mal-rotation | Yes | Luminal resection |
| G5020 | Robotic assisted laparoscopic switch of duodenum with vertical gastrectomy and biliary pancreatic diversion | Yes | Luminal resection |
| G5050 | Endoscopic submucosal dissection of duodenal lesions | No | Endoscopy |
| G5100 | Bypass of duodenum | Yes | Luminal resection |
| G5320 | Closure of perforated ulcer of duodenum | No | No luminal resection |
| G5810 | Excision of jejunum | Yes | Luminal resection |
| G6000 | Open formation of jejunostomy | No | No luminal resection |
| G6080 | Laparoscopically assisted resection of small intestine | Yes | Luminal resection |
| G6082 | Open resection of small intestine tumour | Yes | Luminal resection |
| G6100 | Bypass of jejunum | Yes | Luminal resection |
| G6500 | Diagnostic oesophago-gastro-duodenoscopy (OGD) includes forceps biopsy, biopsy urease test and dye spray | No | Endoscopy |
| G6710 | Intubation of jejunum for decompression of intestine (without laparotomy) | No | No luminal resection |
| G7100 | Bypass of ileum | Yes | Luminal resection |
| G7250 | Ileoanal anastomosis and creation of pouch | Yes | Luminal resection |
| G7402 | Open formation of ileostomy | No | No luminal resection |
| G7403 | Laparoscopic ileostomy | No | No luminal resection |
| G7512 | Revision of ileostomy - local | No | No luminal resection |
| G7513 | Revision of ileostomy - laparotomy | Yes | Luminal resection |
| G7530 | Closure of ileostomy (as sole procedure) | No | No luminal resection |
| G7900 | Ileoscopy via stoma with therapy | No | Endoscopy |
| G8080 | Small bowel capsule endoscopy (including interpretation and evaluation) | No | Endoscopy |
| G8081 | Upper GI capsule endoscopy (including interpretation and evaluation) | No | Endoscopy |
| G8082 | Diagnostic oesophago-gastro-duodenoscopy (OGD) and immediate colonoscopy includes forceps biopsies, biopsy test and dye spray (as sole procedure) | No | Endoscopy |
| G8083 | Therapeutic oesophago-gastro-duodenoscopy (OGD) and immediate colonoscopy includes forceps biopsies, biopsy test and dye spray (as sole procedure) | No | Endoscopy |
| G8084 | Diagnostic oesophago-gastro-duodenoscopy (OGD) and immediate flexible sigmoidoscopy includes forceps biopsies, biopsy test and dye spray (as sole procedure) | No | Endoscopy |
| G8085 | Therapeutic oesophago-gastro-duodenoscopy (OGD) and immediate flexible sigmoidoscopy includes forceps biopsies, biopsy test and dye spray (as sole procedure) | No | Endoscopy |
| G8086 | Colon capsule endoscopy (including interpretation and evaluation) | No | Endoscopy |
| G8090 | Small bowel physiology studies including manometry | No | Investigation |
| H0210 | Appendicectomy | No | No luminal resection |
| H0280 | Laparoscopic appendicectomy | No | No luminal resection |
| H0310 | Drainage of abscess of appendix or drainage of intra-abdominal abscess | No | No luminal resection |
| H0410 | Panproctocolectomy and ileostomy | Yes | Luminal resection |
| H0480 | Abdominal revision of restorative proctocolectomy | Yes | Luminal resection |
| H0510 | Total excision of colon and ileorectal anastomosis | Yes | Luminal resection |
| H0610 | Extended excision of right hemicolon | Yes | Luminal resection |
| H0700 | Right hemicolectomy | Yes | Luminal resection |
| H0750 | Laparoscopically assisted right hemicolectomy | Yes | Luminal resection |
| H0800 | Excision of transverse colon | Yes | Luminal resection |
| H0900 | Excision of left hemicolon | Yes | Luminal resection |
| H1000 | Excision of sigmoid colon | Yes | Luminal resection |
| H1200 | Excision of lesion of colon (transabdominal) | No | No luminal resection |
| H1300 | Bypass of colon | Yes | Luminal resection |
| H1542 | Closure of colostomy | No | No solid organ resection |
| H1550 | Abdominal operation for Hirschsprung's disease (e.g. Duhamel, Söave and Surcuson operations) | No | Paediatric procedure |
| H1581 | Laparoscopic colostomy and stoma formation (including revision) | No | No luminal resection |
| H1590 | Open formation of colostomy | No | No luminal resection |
| H1700 | Intra abdominal manipulation of colon for intussusception (as sole procedure) | No | Paediatric procedure |
| H1880 | Laparoscopically assisted left colon resection | Yes | Luminal resection |
| H2001 | Double Balloon Enteroscopy | No | Endoscopy |
| H2002 | Diagnostic colonoscopy, includes forceps biopsy of colon and ileum | No | Endoscopy |
| H2003 | Therapeutic colonoscopy with snare loop biopsy or excision of lesion | No | Endoscopy |
| H2180 | Fibreoptic colonoscopy and recanalisation of tumour | No | Endoscopy |
| H2220 | Endoscopic ultrasound for tumour staging, including diagnostic endoscopy | No | Investigation |
| H2280 | CT colonography (virtual colonoscopy) | No | Investigation |
| H2350 | Endoscopic mucosal resection (EMR) of colorectal polyp | No | Endoscopy |
| H2380 | Endoscopic submucosal dissection (ESD) of colorectal polyp | No | Endoscopy |
| H2502 | Diagnostic flexible sigmoidoscopy, including forceps biopsy and proctoscopy | No | Endoscopy |
| H2503 | Therapeutic sigmoidoscopy with snare loop biopsy or excision of lesion | No | Endoscopy |
| H2510 | Rigid sigmoidoscopy including proctoscopy and biopsy | No | Endoscopy |
| H3310 | Abdominoperineal pull through resection with colo-anal anastomosis +/- colonic pouch and associated stoma | Yes | Luminal resection |
| H3320 | Abdominoperineal resection of rectum and anus | Yes | Luminal resection |
| H3322 | Laparoscopic abdominoperineal resection | Yes | Luminal resection |
| H3332 | Anterior resection - high (i.e. colorectal anastomosis above the peritoneal reflection) | Yes | Luminal resection |
| H3334 | Anterior resection - low (i.e. colorectal anastomosis at or below the peritoneal reflection) | Yes | Luminal resection |
| H3335 | Endoscopic vacuum therapy for colorectal anastomotic leakage | No | Endoscopy |
| H3362 | Hartmann's procedure | Yes | Luminal resection |
| H3363 | Colectomy and colostomy and preservation of rectum | Yes | Luminal resection |
| H3364 | Laparoscopic anterior resection - high (i.e. colorectal anastomosis above the peritoneal reflection) | Yes | Luminal resection |
| H3365 | Laparoscopic anterior resection - low (i.e. colorectal anastomosis at or below the peritoneal reflection) | Yes | Luminal resection |
| H3380 | Partial excision of rectum and sigmoid colon for prolapse | Yes | Luminal resection |
| H3382 | Proctectomy | Yes | Luminal resection |
| H3381 | Total Mesorectal Excision | Yes | Luminal resection |
| H3383 | Robotic assisted laparoscopic total mesorectal excision (TME) | Yes | Luminal resection |
| H3384 | Open Total Mesorectal Excision (TME) | Yes | Luminal resection |
| H3385 | Laparoscopic Total Mesorectal Excision (TME) | Yes | Luminal resection |
| H3386 | Transanal Total Mesorectal Excision (TME) | Yes | Not intra-peritoneal procedure |
| H3390 | Reversal of Hartmann's procedure | Yes | Luminal resection |
| H3400 | Open excision of lesion of rectum and colon | Yes | Luminal resection |
| H3500 | Fixation of rectum for prolapse | No | No luminal resection |
| H3580 | Laparoscopic rectopexy without mesh | No | No luminal resection |
| H3581 | Robotic assisted laparoscopic rectopexy | No | No luminal resection |
| H3582 | Laparoscopic ventral mesh rectopexy (LVMR) | No | No luminal resection |
| H3590 | Stapled transanal rectal resection (STARR) for obstructed defaecation syndrome | No | No luminal resection |
| H4000 | Transanal resection for rectal cancer | No | Not intra-peritoneal procedure |
| H4050 | Endoscopic radiofrequency therapy of the anal sphincter for faecal incontinence | No | Endoscopy |
| H4080 | Injection of bulking agents for faecal incontinence | No | No luminal resection |
| H4122 | Transanal endoscopic microsurgery | No | Endoscopy |
| H4130 | Perianal excision of lesion of rectum (including sigmoidoscopy) | No | Not intra-peritoneal procedure |
| H4180 | Full or partial thickness rectal biopsy | No | Not intra-peritoneal procedure |
| H4190 | Therapeutic High Resolution Anoscopy (HRA) in symptomatic patients (+/- biopsy or ablation of lesion of anus) | No | Endoscopy |
| H4200 | Perineal repair of prolapse of rectum | No | No luminal resection |
| H4420 | Faecal disimpaction | No | No luminal resection |
| H4430 | Examination of rectum under anaesthetic (as sole procedure) | No | No luminal resection |
| H4480 | Dilation of stricture of rectum | No | No luminal resection |
| H4680 | Repair of faecal fistula | No | No luminal resection |
| H4800 | Excision of lesion of anus | No | No luminal resection |
| H4900 | Destruction of lesion of anus | No | No luminal resection |
| H5020 | Repair of anal sphincter (including sigmoidoscopy) | No | No luminal resection |
| H5042 | Primary repair of high/intermediate congenital ano-rectal anomaly | No | No luminal resection |
| H5043 | Primary repair of low congenital ano-rectal anomaly | No | No luminal resection |
| H5080 | Repair of anal trauma | No | No luminal resection |
| H5100 | Haemorrhoidectomy (including sigmoidoscopy) | No | No luminal resection |
| H5101 | Laser haemorrhoidectomy (including sigmoidoscopy) | No | No luminal resection |
| H5120 | Radiofrequency ablation of haemorrhoids | No | No luminal resection |
| H5230 | Injection of sclerosing substance into haemorrhoids | No | No luminal resection |
| H5240 | Banding of haemorrhoids | No | No luminal resection |
| H5250 | Circular Stapling Haemorrhoidectomy | No | No luminal resection |
| H5260 | Low voltage treatment of internal haemorrhoids | No | No luminal resection |
| H5400 | Anorectal stretch | No | No luminal resection |
| H5510 | Laying open of low anal fistula (fistulotomy) (including sigmoidoscopy) | No | No luminal resection |
| H5520 | Laying open of high anal fistula (fistulotomy) (including sigmoidoscopy) | No | No luminal resection |
| H5530 | Closure of anal fistula using a suturable bioprosthetic or synthetic plugs | No | No luminal resection |
| H5540 | Seton placement for treatment of anal fistula | No | No luminal resection |
| H5541 | Adjustment or removal of Seton under general anaesthetic | No | No luminal resection |
| H5560 | Ligation of the intersphincteric fistula tract (LIFT) for the treatment of anal fistula | No | No luminal resection |
| H5561 | Endoscopic ablation for an anal fistula +/- flap | No | No luminal resection |
| H5562 | Collagen paste for closing an anal fistula | No | No luminal resection |
| H5580 | Endoscopic ablation for an anal fistula without flap | No | No luminal resection |
| H5620 | Lateral sphincterotomy of anus | No | No luminal resection |
| H5640 | Excision of anal fissure | No | No luminal resection |
| H5800 | Drainage through perineal region (including ischio-rectal abscess) (including sigmoidoscopy) | No | No luminal resection |
| H5940 | Excision of pilonidal sinus and suture/skin graft | No | No luminal resection |
| H5941 | Excision of pilonidal sinus with flap reconstruction | No | No luminal resection |
| H6020 | Laying open of pilonidal sinus | No | No luminal resection |
| H6050 | Endoscopic ablation for a pilonidal sinus | No | No luminal resection |
| H6260 | Proctoscopy (+/- biopsy) | No | Endoscopy |
| H6280 | Faecal microbiota transplant for the treatment of recurrent clostridium difficile Infection | No | No luminal resection |
| H6840 | Flexible pouchoscopy +/- biopsy and/or removal of polyp(s) | No | Endoscopy |
| J0200 | Partial hepatectomy (left hepatectomy or resection of up to three segments) +/- cholecystectomy | Yes | Resection of solid organ |
| J0210 | Hemihepatectomy (resection of four or more segments) +/- cholecystectomy | Yes | Resection of solid organ |
| J0220 | Robotic assisted hemihepatectomy | Yes | Resection of solid organ |
| J0310 | Resection of liver tumour | Yes | Resection of solid organ |
| J0312 | Microwave ablation for primary or metastatic cancer of the liver | No | No solid organ resection |
| J0400 | Repair of liver (including therapeutic laparoscopic operations on liver) | No | No solid organ resection |
| J0510 | Open drainage of liver | No | No solid organ resection |
| J0740 | Open hepatectomy and ablation | Yes | Resection of solid organ |
| J0780 | Radiofrequency thermocoagulation of liver with scalpel liver resection | No | No solid organ resection |
| J0781 | Chemosaturation therapy for primary or metastatic cancer of the liver | No | No solid organ resection |
| J0900 | Diagnostic laparoscopy (including any biopsy) | No | No luminal resection |
| J1041 | Hepatic venous wedge pressure (HVWP) | No | No solid organ resection |
| J1140 | TIPS Stent tipsogram | No | No solid organ resection |
| J1300 | Percutaneous biopsy of lesion of liver | No | Not intra-peritoneal procedure |
| J1400 | Open puncture of liver | No | No solid organ resection |
| J1800 | Cholecystectomy (including mini-cholecystectomy) | No | No solid organ resection |
| J1820 | Cholecystectomy with exploration of common bile duct | No | No solid organ resection |
| J1830 | Laparoscopic cholecystectomy | No | No solid organ resection |
| J1831 | Robotic assisted Laparoscopic cholecystectomy | No | No solid organ resection |
| J1880 | Laparoscopic cholecystectomy with peri-operative cholangiogram | No | No solid organ resection |
| J1900 | Anastomosis of gall bladder (to another viscus) | No | No solid organ resection |
| J2720 | Partial excision of bile duct and anastomosis of bile duct to duodenum/jejunum | Yes | Luminal resection |
| J2800 | Excision of lesion of bile duct | No | No luminal resection |
| J2900 | Anastomosis of hepatic duct | Yes | Luminal resection |
| J3000 | Anastomosis of common bile duct | Yes | Luminal resection |
| J3100 | Open introduction of prosthesis into bile duct | No | No luminal resection |
| J3200 | Repair of bile duct | Yes | Luminal resection |
| J3300 | Incision of bile duct (including exploration for calculus removal) | No | No luminal resection |
| J3500 | Sphincterotomy of bile duct and pancreatic duct using duodenal approach | No | No luminal resection |
| J3800 | Therapeutic ERCP without insertion of stents | No | Endoscopy |
| J3900 | Therapeutic ERCP with insertion of biliary or pancreatic stent(s), sphincterotomy or stone extraction | No | Endoscopy |
| J4300 | Diagnostic ERCP ( includes forceps biopsy) | No | Endoscopy |
| J5000 | Percutaneous examination of bile duct | No | Not intra-peritoneal procedure |
| J5480 | Pancreatic transplant including sequential pancreatic transplant | No | Transplant procedure |
| J5481 | Pancreatic with kidney transplant (simultaneous pancreas SPK) | No | Transplant procedure |
| J5500 | Total pancreatectomy and excision of surrounding tissue | Yes | Resection of solid organ |
| J5520 | Total pancreatectomy | Yes | Resection of solid organ |
| J5610 | Pancreatoduodenectomy and excision of surrounding tissue (Whipple's procedure) | Yes | Resection of solid organ |
| J5611 | Laparoscopic pancreatoduodenectomy and excision of surrounding tissue (Whipple's procedure) | Yes | Resection of solid organ |
| J5612 | Robotic-assisted pancreatoduodenectomy and excision of surrounding tissue (Whipple's procedure) | Yes | Resection of solid organ |
| J5700 | Distal pancreatectomy | Yes | Resection of solid organ |
| J5711 | Pancreatectomy with autologous islet cell transplantation | No | Transplant procedure |
| J5712 | Robotic assisted distal pancreatectomy | Yes | Resection of solid organ |
| J5750 | Laparascopic distal pancreatectomy | Yes | Resection of solid organ |
| J5800 | Excision of lesion of pancreas | Yes | Resection of solid organ |
| J5810 | Irreversible electroporation of pancreatic tumors | No | No solid organ resection |
| J5900 | Anastomosis of pancreatic duct (to another viscus) | Yes | Luminal resection |
| J6100 | Open drainage of lesion of pancreas | Yes | Resection of solid organ |
| J6180 | Drainage of pancreatic abscess | No | No solid organ resection |
| J6200 | Incision of pancreas | No | No solid organ resection |
| J6300 | Open examination of pancreas | No | No solid organ resection |
| J6600 | Therapeutic percutaneous operations on pancreas | No | Not intra-peritoneal procedure |
| J6730 | Endoscopic upper gastrointestinal ultrasound, e.g. for pancreatico-biliary diagnosis/transmucosal biopsy | No | Endoscopy |
| J6900 | Open splenectomy | No | Not associated with GI tract |
| J6980 | Laparoscopic splenectomy | No | Not associated with GI tract |
| J9901 | Cytoreductive surgery (Sugarbaker technique) for Pseudomyxoma Peritonei with intraperitoneal chemotherapy | Yes | Luminal resection |
| J9902 | Cytoreductive surgery for Colorectal Peritoneal Carcinomatosis (2-3 distinct procedures) with intraperitoneal chemotherapy | Yes | Luminal resection |
| J9903 | Cytoreductive surgery for Colorectal Peritoneal Carcinomatosis (4-6 distinct procedures) with intraperitoneal chemotherapy | Yes | Luminal resection |
| J9904 | Cytoreductive surgery for Colorectal Peritoneal Carcinomatosis (7-8 distinct procedures) with intraperitoneal chemotherapy | Yes | Luminal resection |
| J9905 | Repeat Cytoreductive surgery for Pseudomyxoma Peritonei or Colorectal Peritoneal Carcinomatosis with intraperitoneal chemotherapy | Yes | Luminal resection |
| J9906 | Heated intraperitoneal chemotherapy for Colorectal Peritoneal Carcinomatosis | No | No luminal resection |
| J9907 | Cytoreductive surgery for Ovarian Malignancies excluding intraperitoneal chemotherapy | Yes | Luminal resection |
| J9908 | Cytoreductive surgery for stage III epithelial ovarian malignancies including Heated Intraperitoneal Chemotherapy (HIPEC) | Yes | Luminal resection |
| L7032 | Haemorrhoidal artery ligation operation (including image-guided) +/- recto anal prolapse repair | No | No luminal resection |
| L7040 | Open cannulation of intra abdominal artery for infusion chemotherapy | No | No luminal resection |
| L7710 | Creation of portocaval shunt | No | No luminal resection |
| L7712 | Inferior vena cavogram | No | No luminal resection |
| L7920 | Plication of vena cava | No | No luminal resection |
| L7980 | Repair of wound of major artery or vein of abdomen (including aorta and vena cava) | No | No luminal resection |
| L8110 | Creation of peritoneo-venous shunt (Levine/Denver) | No | No luminal resection |
| T1640 | Repair of congenital diaphragmatic hernia | No | No luminal resection |
| T1900 | Simple excision of inguinal hernial sac (herniotomy) - unilateral | No | No luminal resection |
| T1910 | Simple excision of inguinal hernial sac (herniotomy) - bilateral | No | No luminal resection |
| T2000 | Primary repair of inguinal hernia | No | No luminal resection |
| T2002 | Laparoscopic repair of inguinal hernia - unilateral | No | No luminal resection |
| T2003 | Repair of inguinal hernia requiring removal of previously inserted mesh | No | No luminal resection |
| T2010 | Primary repair of inguinal hernia - bilateral | No | No luminal resection |
| T2012 | Laparoscopic repair of inguinal hernia - bilateral | No | No luminal resection |
| T2080 | Primary repair of strangulated inguinal hernia | No | Not intra-peritoneal procedure |
| T2100 | Repair of recurrent inguinal hernia | No | No luminal resection |
| T2102 | Laparoscopic repair of recurrent inguinal hernia - unilateral | No | No luminal resection |
| T2110 | Repair of recurrent inguinal hernia - bilateral | No | No luminal resection |
| T2112 | Laparoscopic repair of recurrent inguinal hernia - bilateral | No | No luminal resection |
| T2200 | Primary repair of femoral hernia | No | No luminal resection |
| T2203 | Repair of femoral hernia requiring removal of previously inserted mesh | No | No luminal resection |
| T2280 | Primary repair of strangulated femoral hernia | No | Not intra-peritoneal procedure |
| T2300 | Repair of recurrent femoral hernia | No | No luminal resection |
| T2400 | Repair of umbilical/paraumbilical hernia (irrespective of age) | No | No luminal resection |
| T2403 | Repair of umbilica/paraumbilical hernia requiring removal of previously inserted mesh | No | No luminal resection |
| T2500 | Open repair of incisional hernia not requiring mesh | No | No luminal resection |
| T2501 | Open repair of incisional hernia requiring mesh | No | No luminal resection |
| T2503 | Laparoscopic repair of incisional hernia not requiring mesh | No | No luminal resection |
| T2510 | Laparoscopic repair of parastomal hernia requiring mesh | No | No luminal resection |
| T2600 | Repair of recurrent incisional hernia not requiring mesh | No | No luminal resection |
| T2620 | Repair of recurrent incisional hernia requiring mesh | No | No luminal resection |
| T2640 | Repair of recurrent incisional hernia requiring removal of previously inserted mesh | No | No luminal resection |
| T2720 | Laparoscopic repair of incisional hernia requiring mesh | No | No luminal resection |
| T2730 | Repair of dorsal hernia including lumbar hernia | No | No luminal resection |
| T2740 | Repair of perineal hernia including scrotal that are not inguinal | No | No luminal resection |
| T2750 | Repair of sciatic hernias | No | No luminal resection |
| T2761 | Laparoscopic repair of Spigelian hernia with mesh | No | No luminal resection |
| T2762 | Open repair of Spigelian hernia with mesh | No | No luminal resection |
| T2763 | Laparoscopic repair of Spigelian hernia without mesh | No | No luminal resection |
| T2764 | Open repair of Spigelian hernia without mesh | No | No luminal resection |
| T2780 | Open Component Separation Technique (CST) repair for complex abdominal hernia with mesh | No | No luminal resection |
| T2781 | Repair of epigastric hernia | No | No luminal resection |
| T2782 | Minimally invasive Component Separation Technique (CST) repair for complex abdominal hernia with mesh | No | No luminal resection |
| T2783 | Open Component Separation Technique (CST) repair for complex abdominal hernia without mesh | No | No luminal resection |
| T2784 | Minimally invasive Component Separation Technique (CST) repair for complex abdominal hernia without mesh | No | No luminal resection |
| T2830 | Resuture of previous incision in abdominal wall (burst abdomen) | No | No luminal resection |
| T3010 | Laparotomy for post-operative haemorrhage | No | No luminal resection |
| T3080 | Laparotomy and repair of multiple visceral trauma | Yes | Intra-peritoneal procedure |
| T3410 | Open drainage of subphrenic abscess | No | No luminal resection |
| T3600 | Wedge excision or removal of omentum (as sole procedure) | No | No solid organ resection |
| T3610 | Omental biopsy +/- an ascitic drain under image guidance | No | No solid organ resection |
| T3910 | Excision of retroperitoneal tumour, +/-ureterolysis | Yes | Luminal resection |
| T3920 | Multivisceral resection of retroperitoneal sarcoma | Yes | Luminal resection |
| T3930 | Surgical drainage of retroperitoneal abscess | No | No luminal resection |
| T3980 | Excision of presacral tumour | Yes | Luminal resection |
| T3990 | Excision of retroperitoneal neuro-endocrine lesion | No | No luminal resection |
| T4130 | Freeing of adhesions of peritoneum | No | No luminal resection |
| T4300 | Laparoscopic adhesiolysis (including biopsy) | No | No luminal resection |
| T4302 | Open adhesiolysis (including biopsy) | No | No luminal resection |
| T4610 | Paracentesis abdominis for ascites | No | No luminal resection |
| T4680 | Suprapubic drainage of pelvic abscess | No | No luminal resection |
| T7972 | Exploration and repair of groin disruption including repair of muscle fascia and tendors (Gilmore's groin repair) | No | No luminal resection |
| XR330 | Gastric intubation under imaging control (as sole procedure) | No | Endoscopy |
| X1410 | Total exenteration of pelvis | Yes | Luminal resection |
| X1430 | Posterior exenteration of pelvis | Yes | Luminal resection |
| J9908 | Cytoreductive surgery for stage III epithelial ovarian malignancies including Heated Intraperitoneal Chemotherapy (HIPEC) | Yes | Luminal resection |

## Table S2. Reasons for allocation to non-MAS category or exclusion of CCSD codes.

| Reason for exclusion | Number of CCSD codes |
| --- | --- |
| Non-MAS category |  |
| No luminal resection | 146 |
| No solid organ resection | 21 |
| Not intra-peritoneal procedure | 10 |
| Not associated with GI tract | 8 |
| Involvement of thorax | 4 |
| Paediatric procedure | 4 |
| Transplant procedure | 3 |
| Excluded |  |
| Endoscopy | 46 |
| Investigation | 7 |
| Interventional radiology | 0 |
| Radiotherapy | 5 |

**Table S3. Confusion matrix comparing human and machine classification of major abdominal surgery. Expert reviewer result represents translation of narrative definition into procedure code.**

|  |  | **PAM clustering result** | |
| --- | --- | --- | --- |
|  |  | **1 (MAS)** | **2 (non-MAS)** |
| **Expert reviewer result** | **MAS** | **42** | **7** |
|  | **non-MAS** | **9** | **69** |

**Table S4. Discordance between narrative definition and unsupervised machine learning clustering.**

|  | MAS | Reason for inclusion in / exclusion from MAS definition |
| --- | --- | --- |
|  | **Narrative MAS but clustered as non-MAS by unsupervised machine learning** | |
| Partial gastrectomy | Yes | Luminal resection |
| Sleeve gastrectomy (including laparoscopic) | Yes | Luminal resection |
| Gastric bypass eg. Roux en Y for morbid obesity (including laparoscopic) | Yes | Luminal resection |
| Laparoscopic biliary gastric bypass | Yes | Luminal resection |
| Bypass of duodenum | Yes | Luminal resection |
| Laparoscopically assisted resection of small intestine | Yes | Luminal resection |
| Cytoreductive surgery for Ovarian Malignancies excluding intraperitoneal chemotherapy | Yes | Luminal resection |
|  | **Narrative non-MAS but clustered as MAS by unsupervised machine learning** | |
| Drainage of abscess of appendix or drainage of intra-abdominal abscess | No | No luminal resection |
| Open formation of colostomy | No | No luminal resection |
| Intra abdominal manipulation of colon for intussusception (as sole procedure) | No | No luminal resection |
| Fixation of rectum for prolapse | No | No luminal resection |
| Open Component Separation Technique (CST) repair for complex abdominal hernia with mesh | No | No luminal resection |
| Resuture of previous incision in abdominal wall (burst abdomen) | No | No luminal resection |
| Laparotomy for post-operative haemorrhage | No | No luminal resection |
| Chemosaturation therapy for primary or metastatic cancer of the liver | No | No solid organ resection |
| Adrenalectomy - unilateral (open) | No | Not associated with GI tract |

**References**

1. cluster: Cluster Analysis Basics and Extensions. https://CRAN.R-project.org/package=cluster.
